# Recognition of an Intracranial Meningioma in a Woman With Worsening Headache Presenting to a Chiropractor: A Case Report

**DOI:** 10.7759/cureus.97462

**Published:** 2025-11-21

**Authors:** Gina N Zamary, Robert J Trager

**Affiliations:** 1 Chiropractic, Private Practice, Cleveland, USA; 2 Chiropractic, Connor Whole Health, University Hospitals Cleveland Medical Center, Cleveland, USA; 3 Family Medicine and Community Health, Case Western Reserve University School of Medicine, Cleveland, USA

**Keywords:** case report, chiropractic, examination, headache, meningioma, neurology

## Abstract

Meningiomas are the most common primary intracranial tumors in adults. They are typically slow-growing and often present with nonspecific symptoms such as headache and neck pain, which may overlap with common musculoskeletal complaints encountered in chiropractic practice. A 41-year-old woman presented to a chiropractor with a two-year history of worsening neck pain, headaches, upper extremity paresthesia, and subjective balance disturbance. Examination revealed hyperreflexia and bilateral Hoffman’s reflexes. Given these upper motor neuron lesion signs, the chiropractor ordered cervical spine radiographs, which were normal, followed by magnetic resonance imaging, which revealed a large posterior fossa mass consistent with a meningioma. The patient was referred for neurosurgical management, underwent successful surgical resection, and was scheduled for adjuvant radiation therapy. This case illustrates how patients with serious intracranial pathology may present to a chiropractor in cases where symptoms appear musculoskeletal. Considering meningiomas are slow-growing, their clinical signs may be subtle. Careful neurological examination and recognition of red flags, even when radiographs are normal, are critical for timely referral and diagnosis.

## Introduction

Meningiomas are the most common primary intracranial tumors in adults, account for 40.8% of all primary brain tumors in the United States (US), and have an incidence of 9.7 per 100,000 person-years [1]. They occur about twice as often in women compared with men, and the median age at diagnosis is 66 years [1,2]. Risk factors for meningiomas include ionizing radiation exposure, obesity, hormone replacement therapy use, and occupational herbicide exposure [2,3]. Although intracranial tumors can cause a variety of symptoms depending on location, many meningiomas remain clinically silent until they grow large enough to produce headaches, neck pain, or other nonspecific complaints [1,4]. Specifically, meningiomas cause symptoms due to mass effect on brain tissue, obstruction of cerebrospinal fluid, and/or pressure on neurovascular structures [1,4]. Other signs and symptoms may include focal cranial nerve deficit, seizure, cognitive change, weakness, vertigo/dizziness, ataxia/gait change, pain/sensory change, proptosis, and syncope [2]. Despite the benign reputation of meningiomas, the signs and symptoms associated with the tumor can significantly impact a person’s quality of life.

A provisional diagnosis of meningioma can be made via magnetic resonance imaging (MRI) or contrast-enhanced computed tomography, but a tissue sample is needed to confirm the diagnosis and rule out other diagnoses, such as metastasis [2,5]. Management of a meningioma is highly individualized and can include observation, surgical resection, radiotherapy, or rarely, chemotherapy [2]. Observation is the first option for incidental or asymptomatic meningiomas, while surgical resection is the first option for symptomatic or growing meningiomas [6,7]. When observation is indicated, follow-up MRI is recommended annually for five years [6]. The goal of surgery is to have a gross total resection of the tumor. In cases in which this is not possible, complementary therapies, such as radiotherapy, may be required following surgery [6].

As direct-access clinicians, chiropractors frequently evaluate patients with neck pain and headache, complaints that rarely result from potentially serious pathology. Identification of intracranial tumors such as meningioma within chiropractic settings is rarely reported, with few case reports to date [8-10]. Identification of any tumor is also generally rare in chiropractic settings. Limited research has examined the rate at which chiropractors encounter brain tumors, although two studies provided more general estimates for carcinoma and primary tumors. According to a US survey, chiropractors reported encountering one case of undiagnosed carcinoma approximately every eight to nine years of practice [11]. This finding was corroborated by a single cohort study of patients presenting to chiropractors with a new complaint of low back pain, in which two of over 7,000 patients were ultimately diagnosed with a primary tumor [12].

We present the case of a 41-year-old woman with a two-year history of chronic neck pain, headaches, and upper extremity paresthesia, who presented to a chiropractor and was ultimately diagnosed with a meningioma. This report highlights the role of chiropractors in recognizing signs of serious pathology in patients with neck pain and headache.

## Case presentation

A 41-year-old female presented to a chiropractor in 2025, reporting chronic neck pain and lower back pain, and left-sided frontal headaches that occurred one to two times per week. The onset of neck pain started 13 years prior after undergoing a total thyroidectomy for thyroid cancer. After the total thyroidectomy, the patient also underwent radiation therapy for the thyroid tumor. Since then, she has been treated with levothyroxine. The patient reported that the neck pain had gradually worsened over the past two years, while the back pain had remained about the same. The patient rated her neck pain as 7 out of a maximum of 10 using the numeric rating scale. The neck pain primarily affected the left side of her neck and extended into her upper trapezius. She noted several symptoms affecting the left shoulder, including mild weakness, a “bad ache,” and intermittent numbness and tingling in this region. She reported her headaches were mild to moderate in intensity and were worse in the afternoon and evening. She described the quality of the headaches as “tight.”

She denied having dizziness, diplopia, and nausea, yet did endorse photosensitivity associated with the headaches, difficulty with balance, and increased urinary frequency over the past three years, and difficulty finding a comfortable position to sleep. The patient attributed the worsening neck and shoulder pain to her job as an ultrasound technician, a job requiring repetitive movements. She was a former smoker and had no family history of neurological disorders or malignancy. The patient had previously undergone physical therapy treatment focused on the neck and shoulder pain, which provided minimal relief from symptoms.

An examination by the chiropractor revealed decreased right cervical rotation with pain, hypertonic and tender upper trapezii and suboccipital muscles. The patient’s upper and lower extremity motor strength was within normal limits. The patient’s muscle stretch reflexes were graded 3+ bilaterally (i.e., increased). Hoffman’s pathologic reflex was present bilaterally (this is an involuntary flexion of the thumb and/or index finger when the examiner flicks the middle finger, suggestive of upper motor neuron lesion). Romberg’s test was normal, and the patient’s gait was within normal limits.

The chiropractor considered the possibility of cervical spinal cord compression due to the hyperreflexia and Hoffman’s reflex, with potential diagnoses including degenerative cervical myelopathy, neoplasm, or disc herniation. Given this differential, the chiropractor avoided any manual thrust manipulations to the cervical spine and instead pursued a diagnostic workup. The chiropractor ordered cervical spine radiographs at the first visit. Apart from the straightening of the cervical lordosis, there were no abnormal findings.

**Figure 1:**
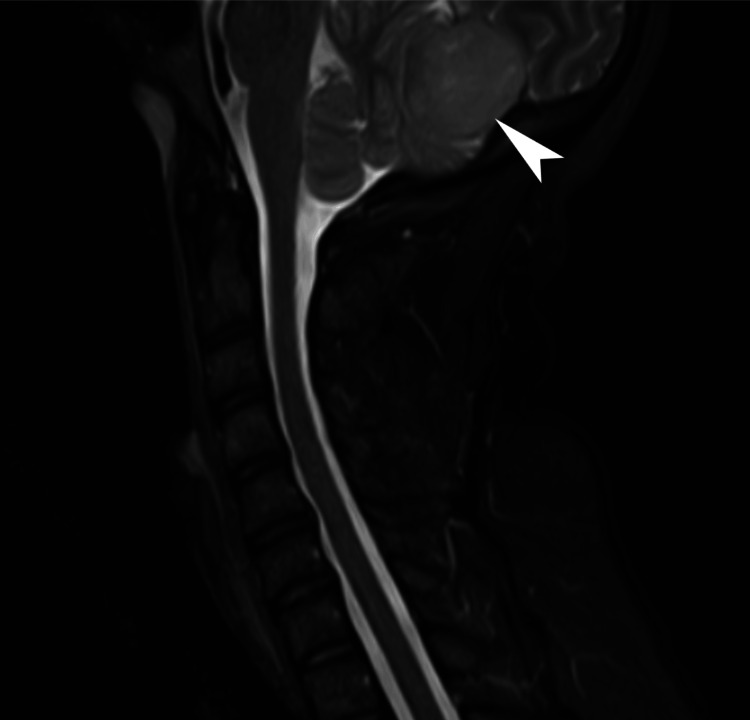
Magnetic resonance imaging of the cervical spine. This mid-sagittal short tau inversion recovery sequence demonstrates an unremarkable cervical spine; however, there is a 3.2 cm posterior fossa mass (arrowhead).

Despite the unremarkable cervical spine radiographs, the chiropractor was still suspicious of cervical spinal cord compression and ordered a cervical spine MRI, which the patient obtained four weeks following the initial chiropractic visit. The radiologist’s impression described a normal cervical spine MRI, but revealed a 3.2 cm extra-axial posterior fossa mass, “statistically most likely to be a meningioma” (Figure 1). The radiologist recommended follow-up MRI of the brain without and with contrast, magnetic resonance venography, and neurosurgical consultation. The patient received the imaging results via her electronic health record and emailed the chiropractor and her primary care provider. The chiropractor discussed the follow-up care with the patient, and the patient’s primary care provider promptly ordered the recommended follow-up brain MRI and magnetic resonance venography and provided a referral to a neurosurgeon.

**Figure 2:**
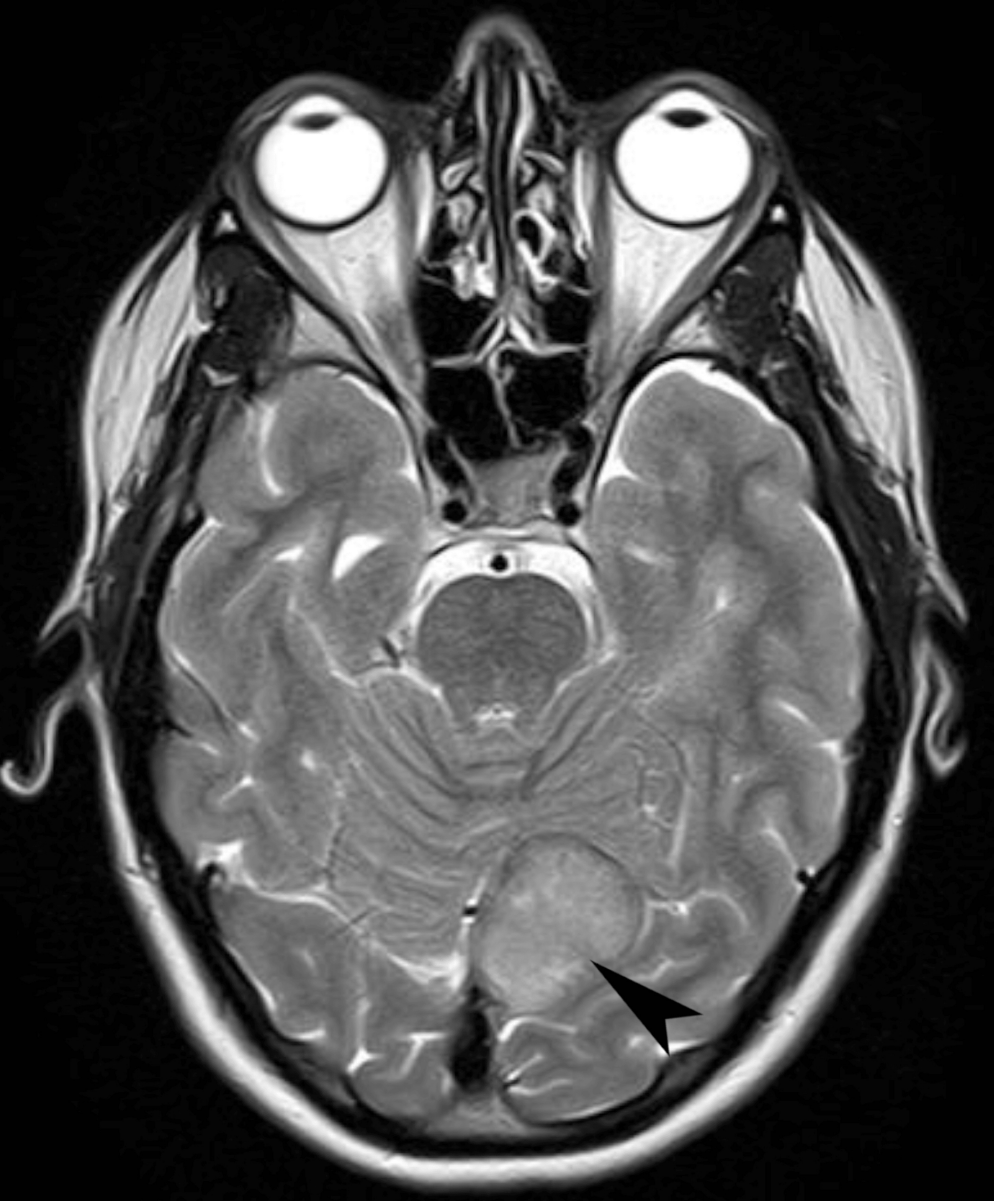
Magnetic resonance imaging of the brain. This axial T2-weighted fast spin echo image further demonstrates the mass (black arrowhead), which is exerting mass effect on the vermis and left cerebellar hemisphere.

Six weeks after the initial chiropractic visit, the patient underwent a brain MRI with and without intravenous gadolinium contrast and brain magnetic resonance venography with and without intravenous gadolinium contrast. The brain MRI revealed a solid 26.6 mm x 33.2 mm extra-axial mass in the posterior fossa on the left side of midline (Figure 2). The imaging revealed that the mass was causing mass effect on the vermis and left cerebellar hemisphere, and slight displacement and mass effect on the inferior sagittal sinus. There was no evidence of hydrocephalus. Magnetic resonance venography revealed that the mass was causing significant narrowing of the medial aspect of the left transverse sinus and the posterior-most aspect of the straight sinus. Magnetic resonance venography also revealed a small sub-centimeter meningioma over the right temporal convexity.

Six weeks after the initial chiropractic visit, the patient also visited a neurosurgeon and was advised to undergo a craniectomy to remove the tumor. Eleven weeks after the neurosurgical consultation, she underwent surgery, and the mass was confirmed to be a transitional meningioma based on biopsy and immunohistochemical study (World Health Organization grade 1: benign). A grade 1 meningioma is defined by its benign nature, low mitotic rate (<4/10 high-power field (HPF)), and no brain invasion [13]. Due to the location of the mass, some of it was unable to be removed. Under the co-management of the surgeon and oncologist, the patient is scheduled for radiation therapy to the surgical area. The neurosurgeon stated that the patient will make a full recovery. At three weeks post-surgery, the chiropractor spoke with the patient, who reported that her recovery was going smoothly. She was able to drive short distances at this time, and will return to work between eight and 12 weeks post-surgery. She had an occupational therapy consult post-op, but was cleared and did not need to complete any ongoing therapy.

## Discussion

The present case highlights a 41-year-old woman diagnosed with a posterior fossa meningioma after presenting to a chiropractor, and she ultimately underwent successful surgical intervention. The clinical features of the present case can be contextualized with the literature. In a survey of 1,852 patients with meningioma, 47% reported a time to diagnosis of six months or less, whereas 18% reported that diagnosis took more than two years [3]. The delay of two to three years from the onset of symptoms in the present case is therefore not unusual and may reflect both the slow growth of meningiomas and their often nonspecific symptoms [2]. The patient’s sex was consistent with the female predominance of meningioma, although her age was younger than the average age at diagnosis, which is in the mid-60s [2].

As exemplified by the present case, headache is a common manifestation of posterior fossa tumors, reported in 70% to 84% of patients, and may result from traction on pain-sensitive structures, mass effect, or increased intracranial pressure [14]. In the present case, there was no evidence of hydrocephalus, infarction, or other distinct primary or secondary headache etiology, suggesting that the most likely mechanism of headache was mass effect from the enlarging posterior fossa lesion.

Notable examination findings included hyperreflexia and bilateral Hoffman’s reflexes, both of which are concerning signs of upper motor neuron lesion. The posterior fossa meningioma was exerting mass effect on the brainstem, where the corticospinal tracts descend, providing a plausible explanation for these abnormal reflexes [15]. The tumor also measured 33 millimeters, which is considered large for meningiomas [1]. While the examination features of meningiomas are variable and dependent on tumor size and location, positive Hoffman’s reflexes have been reported previously among patients with posterior fossa meningioma [15].

The present case also illustrates that meningiomas may present without overt neurological deficits. In a large US series of 11,111 primary brain tumor cases, no neurological sign was detected at the time of diagnosis in 20% of patients with supratentorial tumors and 14.6% of those with infratentorial tumors [16]. The patient’s report of subjective imbalance, although nonspecific, is consistent with this previous study showing that approximately 28% of patients with infratentorial tumors reported incoordination, including unsteady gait or ataxia [16]. While it is difficult to confidently attribute her increased urinary frequency to the meningioma, more severe urinary disturbances, such as incontinence, have been associated with intracranial tumors [14]. Other potential symptoms of meningioma were absent in the present case, including focal cranial nerve deficit, seizure, cognitive change, weakness, and dizziness [2].

Only a handful of case reports have described the diagnosis of brain tumors among patients presenting to chiropractors. One case described a 29-year-old man with a cerebellopontine angle meningioma presenting with neck and upper extremity pain [17]. Another case described a 30-year-old man with a posterior fossa glioma presenting with headaches, gait disturbance, and paresthesia [10]. A third case described a 49-year-old man with a large frontal meningioma who presented with loss of smell and taste [9]. While the current case and two prior cases documented posterior fossa tumors among patients presenting to chiropractors with neck pain and/or headache, the clinical features generally vary, which is expected given the variable presentation of brain tumors.

This case highlights the need for chiropractors to remain alert for subtle neurological red flags in patients with musculoskeletal complaints or headache, including thorough history-taking and examination. Considering meningiomas are slow-growing, neurologic signs such as hyperreflexia or pathologic reflexes may be subtle and/or develop gradually [2]. In this patient, plain radiographs were normal, yet the neurological findings warranted further investigation and led to a diagnosis. Although findings like Hoffman’s can occasionally be seen in healthy individuals (i.e., 2-3% of the general population [18]), their presence alongside unexplained, persistent symptoms should prompt consideration of advanced imaging or referral for further investigation [15].

Limitations

A precise timeline of the development of the patient’s meningioma is unclear, given the lack of historical imaging, the slow growth rate of meningiomas, and the nonspecific, multifactorial nature of her symptoms. In contrast to the headache, the patient’s chronic neck pain is more difficult to attribute directly to the tumor and was potentially multifactorial, considering her occupational strain. Finally, while limited research suggests a positive association between meningioma and a history of thyroid cancer, potentially explained by radiation therapy [19], this potential risk factor cannot be confirmed in the present case. In general, genetic and other environmental influences such as hormone therapy, obesity, and occupational herbicide exposure may also play a role [1,2].

## Conclusions

The present case highlights a 41-year-old woman who presented to a chiropractor and was ultimately diagnosed with a posterior fossa meningioma and underwent successful surgical intervention. The patient’s symptoms, including neck pain, headache, and imbalance, illustrate how intracranial tumors may present with nonspecific features. A careful history and neurological examination were essential, as the combination of upper motor neuron signs and subjective complaints prompted advanced imaging and timely referral. This case reinforces the importance of chiropractors maintaining vigilance for thoroughly evaluating abnormal combinations of symptoms and examination findings.

## References

[1] Wang JZ, Landry AP, Raleigh DR (2024). Meningioma: International Consortium on Meningiomas consensus review on scientific advances and treatment paradigms for clinicians, researchers, and patients. Neuro Oncol.

[2] Ogasawara C, Philbrick BD, Adamson DC (2021). Meningioma: a review of epidemiology, pathology, diagnosis, treatment, and future directions. Biomedicines.

[3] Frey C, Etminan M (2025). Disproportionality analysis of progestogens and estrogens demonstrates increased meningioma risk. J Clin Neurosci.

[4] Nassiri F, Suppiah S, Wang JZ (2020). How to live with a meningioma: experiences, symptoms, and challenges reported by patients. Neurooncol Adv.

[5] Upreti T, Dube S, Pareek V, Sinha N, Shankar J (2024). Meningioma grading via diagnostic imaging: a systematic review and meta-analysis. Neuroradiology.

[6] Goldbrunner R, Stavrinou P, Jenkinson MD (2021). EANO guideline on the diagnosis and management of meningiomas. Neuro Oncol.

[7] Beutler BD, Lee J, Edminster S (2024). Intracranial meningioma: a review of recent and emerging data on the utility of preoperative imaging for management. J Neuroimaging.

[8] Trager RJ, Dusek JA (2021). Chiropractic case reports: a review and bibliometric analysis. Chiropr Man Therap.

[9] Marchand AA, O'Shaughnessy J (2014). Subtle clinical signs of a meningioma in an adult: a case report. Chiropr Man Therap.

[10] Anderson B (2016). Previously undiagnosed malignant brain tumor discovered during examination of a patient seeking chiropractic care. J Chiropr Med.

[11] Daniel DM, Ndetan H, Rupert RL, Martinez D (2012). Self-reported recognition of undiagnosed life threatening conditions in chiropractic practice: a random survey. Chiropr Man Therap.

[12] Chu EC, Trager RJ (2022). Prevalence of serious pathology among adults with low back pain presenting for chiropractic care: a retrospective chart review of integrated clinics in Hong Kong. Med Sci Monit.

[13] Yarabarla V, Mylarapu A, Han TJ, McGovern SL, Raza SM, Beckham TH (2023). Intracranial meningiomas: an update of the 2021 World Health Organization classifications and review of management with a focus on radiation therapy. Front Oncol.

[14] Cho S, Chu MK (2024). Headache in brain tumors. Neurol Clin.

[15] Nadeem A, Khan A, Habib A (2024). Intracranial intricacies: comprehensive analysis of rare skull base meningiomas - a single-center case series. Clin Case Rep.

[16] Zaki A, Natarajan N, Mettlin CJ (1993). Patterns of presentation in brain tumors in the United States. J Surg Oncol.

[17] Stein PJ (2009). A case of cerebellopontine angle meningioma presenting with neck and upper extremity pain. J Manipulative Physiol Ther.

[18] Gruenberger EH, Vatsia SK, Stay RM, Kersey C, Khan MA, Pahl DW (2023). The Hoffmann parallax: a prospective study to determine the benefit of Hoffmann's sign. Orthop Rev (Pavia).

[19] Claus EB, Calvocoressi L, Bondy ML, Schildkraut JM, Wiemels JL, Wrensch M (2011). Family and personal medical history and risk of meningioma. J Neurosurg.

